# Association of Sedentary Behaviour with Metabolic Syndrome: A Meta-Analysis

**DOI:** 10.1371/journal.pone.0034916

**Published:** 2012-04-13

**Authors:** Charlotte L. Edwardson, Trish Gorely, Melanie J. Davies, Laura J. Gray, Kamlesh Khunti, Emma G. Wilmot, Thomas Yates, Stuart J. H. Biddle

**Affiliations:** 1 Diabetes Research Department, University Hospitals of Leicester, Leicester, United Kingdom; 2 School of Sport, Exercise & Health Sciences, Loughborough University, Loughborough, United Kingdom; 3 Department of Cardiovascular Sciences, University of Leicester, Leicester, United Kingdom; 4 Department of Health Sciences, University of Leicester, Leicester, United Kingdom; University of Washington, United States of America

## Abstract

**Background:**

In recent years there has been a growing interest in the relationship between sedentary behaviour (sitting) and health outcomes. Only recently have there been studies assessing the association between time spent in sedentary behaviour and the metabolic syndrome. The aim of this study is to quantify the association between sedentary behaviour and the metabolic syndrome in adults using meta-analysis.

**Methodology/Principal Findings:**

Medline, Embase and the Cochrane Library were searched using medical subject headings and key words related to sedentary behaviours and the metabolic syndrome. Reference lists of relevant articles and personal databases were hand searched. Inclusion criteria were: (1) cross sectional or prospective design; (2) include adults ≥18 years of age; (3) self-reported or objectively measured sedentary time; and (4) an outcome measure of metabolic syndrome. Odds Ratio (OR) and 95% confidence intervals for metabolic syndrome comparing the highest level of sedentary behaviour to the lowest were extracted for each study. Data were pooled using random effects models to take into account heterogeneity between studies. Ten cross-sectional studies (n = 21393 participants), one high, four moderate and five poor quality, were identified. Greater time spent sedentary increased the odds of metabolic syndrome by 73% (OR 1.73, 95% CI 1.55–1.94, p<0.0001). There were no differences for subgroups of sex, sedentary behaviour measure, metabolic syndrome definition, study quality or country income. There was no evidence of statistical heterogeneity (I^2^ = 0.0%, p = 0.61) or publication bias (Eggers test t = 1.05, p = 0.32).

**Conclusions:**

People who spend higher amounts of time in sedentary behaviours have greater odds of having metabolic syndrome. Reducing sedentary behaviours is potentially important for the prevention of metabolic syndrome.

## Introduction

The term metabolic syndrome has been used by researchers to describe the clustering of metabolic risk factors and has been defined by the International Diabetes Federation [1] as central obesity (waist circumference) plus any two of the following four risk factors: raised blood pressure (systolic ≥130 or diastolic ≥85), raised triglycerides (≥150 mg/dL), reduced high density lipoprotein (HDL) cholesterol (<40 mg/dL in males and <50 mg/dL in females) and raised fasting plasma glucose (≥100 mg/dL). Approximately one fourth of European, American and Canadian adults have metabolic syndrome [2]. Previous research has shown that individuals with metabolic syndrome are at an increased risk of diabetes [3], cardiovascular events [4], and mortality from coronary heart disease (CHD), cardiovascular disease (CVD) and all causes [5]. The high prevalence of the syndrome and the associated health consequences demonstrate the importance of understanding the determinants of metabolic syndrome in order to implement prevention strategies.

Sedentary behaviour refers to activities that involve energy expenditure at the level of 1.0–1.5 metabolic equivalent units (METs) [6]. Operationally, sedentary behaviour can be referred to as ‘sitting time’ rather than simply low levels of physical activity. Sedentary behaviour includes activities such as lying down, sitting, watching television, using the computer and other forms of screen-based entertainment. Studies have shown that individuals can spend more than half of their waking hours in sedentary activities [7], [8].

In recent years there has been a growing interest in the relationship between sedentary behaviour and health outcomes. Several recent reviews have highlighted the health risks associated with high sedentary time. For example, sedentary behaviour has been shown to be positively associated with an increased risk of type 2 diabetes [9], [10], cancer [11], and all-cause and CVD mortality [9], [10] and these associations are usually shown to be at least partially independent of levels of physical activity.

Recently there has been an increase in the number of studies assessing the association between time spent in sedentary behaviour and the metabolic syndrome but no reviews on this topic exist. Given the rapid rise in interest in addressing the relationship between sedentary behaviour and health outcomes, and the suggestion that metabolic indicators may be particularly implicated, it is important that evidence is synthesised through a systematic review. Gaps in evidence can then be identified to strengthen the evidence base. The purpose of this research, therefore, is to quantify the association between sedentary behaviour and metabolic syndrome in adults using meta-analysis techniques. This will allow for the assessment of strength and consistency of association, as well as identify any moderators of effect and publication bias. To date, this has not been done for sedentary behaviour and metabolic syndrome.

## Methods

### Search strategy

The study team developed a protocol for the systematic review which is available on request. Medline, Embase and the Cochrane Library were searched up to January 2011. The search strategy included medical subject heading (Mesh) terms related to metabolic syndrome and study designs. The term ‘sedentary lifestyle’ was only recognised as a Mesh term in 2010. To ensure a broad search, a comprehensive list of terms was developed that included the most common forms of sedentary behaviours. Text word, title word, abstract and subject headings were searched in addition to several non-medical subject headings to cover sedentary behaviours and the health outcomes listed. The search strategy can be found in table S1. In addition, the reference lists of articles meeting the inclusion criteria were hand searched along with personal databases for relevant articles.

### Inclusion criteria

To be included in this meta analysis studies had to meet the following criteria: (1) be a cross sectional or prospective design; (2) report data on adults ≥18 years of age; (3) include a self-report or objective measure of time spent sedentary; (4) include an outcome measure of metabolic syndrome; and (5) be published in English. Studies reporting inactivity (i.e., the absence of physical activity) as a measure of sedentariness, rather than a measure of actual time spent in sedentary behaviour, were not included.

Titles and abstracts of identified articles were reviewed independently by CE and EW and the full text of any potentially relevant articles were obtained. If any uncertainty existed, the full text of the article was obtained for discussion between authors (CE and EW). Studies which did not meet the inclusion criteria were disregarded at this stage.

### Data extraction and synthesis

Two authors (CE and TG) independently extracted the data using a data extraction sheet which was developed following procedures recommended by Lipsey and Wilson [12] and Brown, Upchurch and Acton [13]. The following data were extracted for each paper: (1) author, date and country of study; (2) study design; (3) characteristics of study participants (number, age, sex, number with metabolic syndrome); (4) definition and measurement of sedentary behaviour, including any information on reliability and validity; (5) definition and measurement of metabolic syndrome; (6) analysis strategy; and (7) results, including confounders controlled for. The studies employed various measurements of sedentary time (e.g., Television time, total screen (TV, videos and computer) time, sitting time). Furthermore, the measurements of time spent sedentary varied, for example, hours per week, hours per day or hours per day divided into quartiles or arbitrarily divided e.g., <2, 2–3 and ≥3. To overcome this discrepancy in reporting the highest level of sedentary behaviour and the lowest were extracted for each study. Where adjustment for covariates had been made the data were extracted from the most adjusted model. Extraction sheets for each study were cross-checked for consistency, and any discrepancies resolved by discussion.

### Quality assessment

The study team developed a quality assessment tool with reference to MOOSE [14], QATSO [15] and STROBE [16]. The total score available was 7 points (1 point for a prospective study design; if a self-report measure of time spent in sedentary behaviour was used, 1 point for reported validity of the measure, and 1 point for reported reliability of the measure; if an objective measure of time spent in sedentary behaviour was used 2 points; if two or more demographic confounders were controlled for in analyses 1 point; if analyses controlled for physical activity 1 point; if analyses controlled for a measure of weight status 1 point; and 1 point for an objective measure of the health outcome). A score of 6–7 was considered high quality, 4–5 moderate quality, 0–3 poor quality. Two authors (CE and TG) independently assessed all studies for quality.

### Analysis

Odds ratio and 95% confidence intervals comparing the highest level of sedentary behaviour to the lowest were used in the meta-analysis. Where data were reported separately for males and females these were combined using a fixed effects model and the pooled estimate was used, so that each study was included in the main meta-analysis once only. Random effects models were used to pool data because studies were expected to be heterogeneous. Heterogeneity occurs when there is more variation between studies than you would expect by chance [17]. Heterogeneity was assessed using the I^2^ statistic, a measure of the percentage of the variability in effect estimates that is due to heterogeneity rather than sampling error. An I^2^ of over 75% represents considerable heterogeneity [18]. If studies included in a meta analysis are heterogeneous this can affect the validity of the results produced and should be investigated. Forest plots were created, which show the effect estimate, level of variability around that estimate for each study and the weight given to each study in the meta analysis along with the overall pooled result [19]. Publication bias (where studies showing a non significant effect are not published and therefore not included) was assessed visually using contour enhanced funnel plots and the Egger's test (figure S1) [20]. Sub-group analyses for sex, study quality, sedentary behaviour measure, the metabolic syndrome definition employed, and country of study (Western and Australia versus Eastern) were conducted. Differences between subgroups were assessed using meta regression. To assess whether physical activity might confound the relationship between sedentary behaviour and the metabolic syndrome, a sensitivity analysis where we excluded all studies which did not adjust for physical activity (n = 2/10) in their original analysis was conducted and the pooled odds ratios and 95% confidence interval from this analysis were then compared to the analysis that included all studies. All analyses were carried out using Stata (version 11.1). Statistical significance was set at p<0.05 and 95% confidence intervals are quoted throughout.

## Results

### Flow of included studies

**Figure 1:**
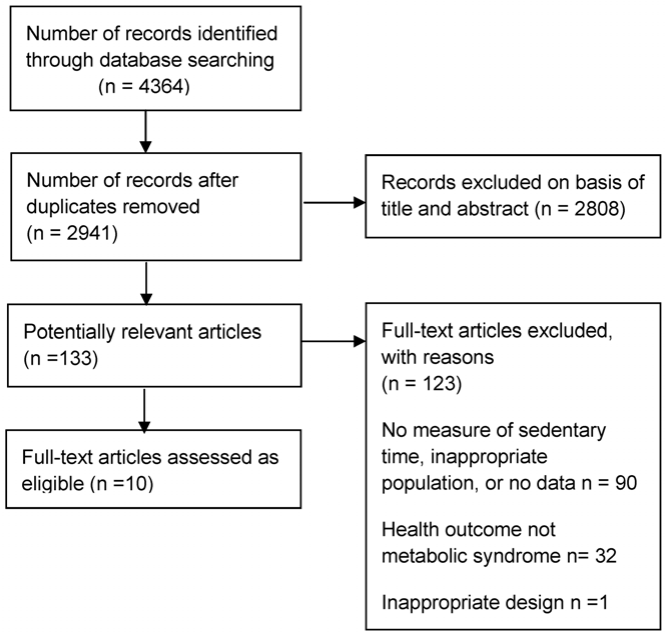
Flow diagram of study selection.

The search identified 4364 articles, from which 133 were identified as potentially relevant. After retrieval of full text, 10 papers were identified that examined the association between sedentary time and metabolic syndrome. One further paper was identified as potentially relevant but subsequently excluded because it reported metabolic syndrome as a continuous risk score rather than grouping participants on the basis of the presence or absence of metabolic syndrome [21]. Figure 1 presents the flow of papers through the study selection process.

### Study characteristics

Study characteristics are shown in Table S2. All 10 studies identified for inclusion were cross-sectional studies. All studies were published between 2005 and 2011. The studies varied in size between 358 and 6162 subjects, with an overall sample size across the studies of 21393. Four studies reported results for men and women combined, four studies reported results for men and women separately, one study reported results for men and women combined and separately, and one study reported results only for women. Four studies assessed self-reported television viewing time (also included video and DVD time), four assessed self-reported leisure total screen time (television and computer use), one study assessed self-reported total sitting time, and one study assessed sedentary time by accelerometry (<100 counts per minute).

### Study quality

None of the studies met all the criteria of the quality assessment score (table S3). Only one study employing a self-report instrument made reference to the reliability or validity of their measure. All studies made adjustments for at least two potential confounding factors, however not all studies adjusted for physical activity (n = 8/10) and body composition. Studies varied in their quality score from 2 to 6 (median 3.5). There was one high quality study, four moderate quality studies, and five poor quality studies.

### Quantitative data synthesis

**Figure 2:**
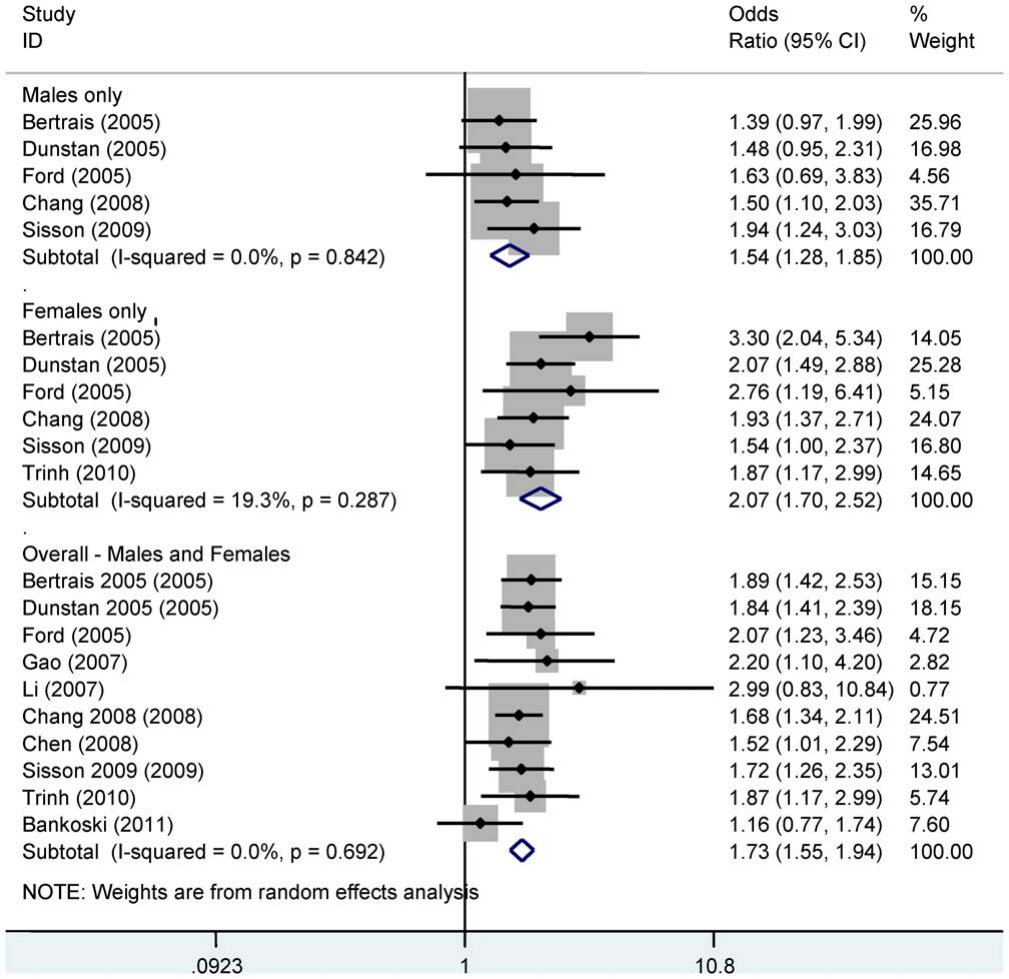
Forest plot for overall results and for the sub-group analysis by sex. The referent group is the lowest sedentary time.

The results for the overall meta-analysis and sub-group analyses are presented in Table S4. Metabolic syndrome was found in 5585 (26.1%) of subjects. The prevalence of metabolic syndrome ranged from 8.9% to 51.6%. Greater time spent sedentary increased the odds of metabolic syndrome by 73% (OR 1.73, 95% CI 1.55–1.94, p<0.0001, Table S4, Figure 2). The results remained largely unchanged after conducting a sensitivity analysis of those studies which adjusted for physical activity (OR 1.73, 95% CI 1.54–1.97, p<0.0001). There were no differences for subgroups of sex, sedentary measure, metabolic syndrome definition, study quality or country income (see Table S4). Sub-group analysis by sex showed that while the increase in odds was greater in females (OR 2.07, CI 1.70–2.52 females vs. 1.54, CI 1.28–1.85 males), the difference between the sexes was not significant (p = 0.24; Table S4, Figure 2).

There was no evidence of statistical heterogeneity (I^2^ = 0.0%, p = 0.61) or publication bias (figure S1; Egger's statistic = 1.05, p = 0.32).

## Discussion

This paper examined the relationship between time spent in sedentary behaviour and the metabolic syndrome using meta-analysis. Results showed that greater time spent sedentary increased the odds of metabolic syndrome by 73%, thus encouraging people to limit their time spent sitting could reduce the risk of metabolic syndrome. This finding is based on 10 cross-sectional studies of which five are of moderate or high quality. The association was not influenced by sex of participants, the sedentary measure or metabolic syndrome definition employed or by study quality. Furthermore, the relationship between sedentary behaviour and the metabolic syndrome may be independent of physical activity, as demonstrated with the sensitivity analysis. This is important because it suggests that sedentary time could be an independent determinant of metabolic dysfunction distinct to that of physical inactivity. This finding is consistent with those reported for other health outcomes, such as all-cause mortality [6], [9]. Moreover, sedentary behaviour, whether measured objectively or subjectively, has been shown to be weakly associated with the amount of time spent in moderate-to-vigorous physical activity [22], [23], confirming one is not simply the inverse of the other. For example, age-adjusted correlation coefficients between TV viewing time and physical activity were as low as −0.11 for women and −0.06 for men in an Australian study [24]. Further work is required on the independent and inter-dependent effects of sedentary and physically active behaviours.

The findings of this meta-analysis are important because metabolic syndrome is a large and growing public health problem [2]. Furthermore, individuals with the metabolic syndrome have been found to have an increased risk of diabetes [25], all cause and cardiovascular disease mortality, an increased incidence of CVD, CHD and stroke compared with individuals who do not have the metabolic syndrome [26].

Investigation of potential mechanisms underpinning the association between sedentary behaviour and metabolic health, although still in its infancy, could explain the association between sedentary time and metabolic syndrome reported here. For example, significant reductions in muscle lipoprotein lipase (LPL) activity, a key enzyme regulating lipid metabolism, have been shown to occur during sedentary activity [27], [28]. Several studies have prevented weight-bearing activity in the hindlimbs of rats and found a substantial reduction in LPL inactivity in skeletal muscles after relatively short periods of immobilisation of the legs [27], [28]; indeed immobilisation has been shown to reduce LPL activity to 10% of its normal function in slow-twitch muscle fibres [29]. These low levels of LPL activity were associated with a large decrease in plasma triglyceride uptake locally in the skeletal muscle, a decrease in HDL cholesterol concentration (approximately 20%) and elevated postprandial lipids [30], [31]. Of note, exercise training was not associated with any increase in LPL activity above control conditions in fast twitch muscle fibres, supporting the notion that reduced sedentary behaviour may have health benefits that are independent to those associated with moderate- to vigorous-intensity physical activity [29]. Although this direct mechanism has not yet been adequately investigated in humans, bed rest studies have confirmed increased sedentary behaviour is associated with a range of deleterious metabolic effects, including deceased lipolysis and marked deteriorations in whole body insulin sensitivity [32], [33], Therefore, although limited in scope, experimental investigation supports the hypothesis that sedentary behaviour may be an independent risk factor for the metabolic syndrome. Sedentary behaviour may also be a risk factor for metabolic syndrome simply on the basis of low energy expenditure resulting in overweight or obesity [34]. Moreover, higher levels of sedentary behaviour are associated with poorer diet [35].

This meta-analysis has several strengths including a broad search on multiple databases, the use of time spent in sedentary behaviour rather than sedentary behaviour as a categorical variable on a physical activity spectrum i.e, defining sedentary behaviour as a lack of physical activity, two independent authors reviewed abstracts and extracted data, and the analysis demonstrated no statistical heterogeneity or publication bias. Nevertheless, the results should be interpreted with some limitations in mind. Most studies (n = 8) used self reported television viewing as the surrogate marker of sitting time and this is a limitation because television viewing may not be a good marker of overall sedentary behaviour, particularly in men [24]. However, if anything, we would expect the use of this maker of sedentary time to weaken the effect because self reporting generally underestimates the amount of time spent sedentary which, in view of the findings, makes a true association between sedentary time and the metabolic syndrome more likely [36]. Future research should aim to measure sedentary behaviour objectively using, for example, accelerometers. A second limitation is that not all studies adjusted for physical activity (n = 8/10) and even though the majority of studies did, physical activity was measured and controlled for in a variety ways (Table S5). For example, some studies entered physical activity in a single step and some in combination with other potential confounders. Finally, all studies in this meta-analysis were cross-sectional, therefore a causal relationship cannot be inferred between sedentary time and metabolic syndrome. Longitudinal and intervention studies are needed to determine the nature of any cause and effect relationship.

In summary, current results, although based on cross-sectional findings, emphasize that it might be important to recommend a reduction in sedentary behaviours, such as TV viewing and time on the computer, for the prevention of metabolic syndrome. However, longitudinal and intervention studies are needed to clarify the nature of any causal relationship between sedentary behaviour and metabolic syndrome.

## Supporting Information

Figure S1 **Contour enhanced funnel plot.**
(DOC)

Table S1 **List of search terms.**
(DOC)

Table S2 **Summary of the studies included in the meta-analysis.**
(DOC)

Table S3 **Quality assessment of the studies included in the meta-analysis.**
(DOC)

Table S4 **Results of the overall meta-analysis, sub-group and sensitivity analysis.**
(DOC)

Table S5 **Summary of physical activity assessment in the studies that controlled for physical activity.**
(DOC)
