# Supplementary material for: Association of Sedentary Behaviour with Metabolic Syndrome: A Meta-Analysis
Source: PLoS One. 2012 Apr 13;7(4):e34916. doi: 10.1371/journal.pone.0034916 (PMC3325927; doi:10.1371/journal.pone.0034916)
Supplement: Figure S1 — Contour enhanced funnel plot. (DOC) [file pone.0034916.s001.doc]

**Figure S1. Contour enhanced funnel plot.**

Egger’s test: test statistic=1.05, p= 0.324
