# Supplementary material for: Association of Sedentary Behaviour with Metabolic Syndrome: A Meta-Analysis
Source: PLoS One. 2012 Apr 13;7(4):e34916. doi: 10.1371/journal.pone.0034916 (PMC3325927; doi:10.1371/journal.pone.0034916)
Supplement: Table S1 — List of search terms. (DOC) [file pone.0034916.s002.doc]

**Table S1. List of search terms.**

| exp SEDENTARY LIFESTYLE/ |
| --- |
| "sedentary".ti,ab |
| "non exercise".ti,ab |
| "non leisure".ti,ab |
| "inactivity".ti,ab |
| "physic* inactiv*".ti,ab |
| "television watch*".ti,ab |
| "TV watch*".ti,ab |
| "television view*".ti,ab |
| "TV view*".ti,ab |
| "screen based".ti,ab |
| "computer use".ti,ab |
| "computer gam*".ti,ab |
| ((screen adj2 time)).ti,ab |
| "couch potato".ti,ab |
| "sitting".ti,ab |
| "car use".ti,ab |
| ((car adj4 driv*)).ti,ab |
| ((car adj4 rid*)).ti,ab |
| "low physical activ*".ti,ab |
| “IPAQ”ti,ab |
| “international physical activity questionnaire”ti,ab |
| exp METABOLIC SYNDROME X/  “metabolic syndrome”.ti.ab |
| ((metabolic adj3 factors)).ti,ab |
| ((clustering adj3 risk factors)).ti,ab |
| “cardio-metabolic”.ti.ab |
| “cardiometabolic”.ti.ab |
| exp CROSS SECTIONAL STUDIES/ |
| cross sectional |
| exp PROSPECTIVE STUDIES/ |
| prospective |
| exp FOLLOW-UP STUDIES/ |
| Follow up |
| exp COHORT STUDIES |
| cohort |
| hazard ratio |
| relative risk |
| observational stud* |
