# Supplementary material for: Association of Sedentary Behaviour with Metabolic Syndrome: A Meta-Analysis
Source: PLoS One. 2012 Apr 13;7(4):e34916. doi: 10.1371/journal.pone.0034916 (PMC3325927; doi:10.1371/journal.pone.0034916)
Supplement: Table S2 — Summary of the studies included in the meta-analysis. (DOC) [file pone.0034916.s003.doc]

**Table S2. Summary of the studies included in the meta-analysis.**

| **Source** | **Country of population** | **N total** | **N without MS** | **N with MS** | **Age range** | **Results reported for^β^** | **Sedentary behaviour measured** | **Metabolic syndrome definition✝** |
| --- | --- | --- | --- | --- | --- | --- | --- | --- |
| Bankoski et al., 2011 [1] | USA | 1367 | 702 | 665 | ≥60y | M&F combined | Accelerometer (time spent at <100cpm) | ATPIII |
| Bertrais et al., 2005 [2] | France | 3834 | 3490 | 344 | 50-69y | M & F separately | TV and computer (average daily time at home) | NCEP |
| Chang et al., 2008 [3] | Taiwan | 2353 | 1570 | 783 | ≥40y | M & F separately | TV (average daily hours) | NCEP* |
| Chen et al., 2008 [4] | China | 1460 | 1160 | 300 | ≥25y | M&F combined | TV, computer and reading (amount of time outside of work) | NCEP* |
| Dunstan et al., 2005 [5] | Australia | 6162 | 5009 | 1153 | >35y | M & F separately | TV/videos (total time in previous week) | WHO |
| Ford et al., 2005 [6] | USA | 1626 | 1102 | 524 | ≥20y | M & F separately and combined | TV/videos and computer (typical day in last month outside of work) | NCEP |
| Gao et al., 2007 [7] | USA (Hispanic population) | 614 | 297 | 317 | ≥60y | M&F combined | TV (average hours per day in past week) | ATPIII |
| Li et al., 2006 [8] | Taiwan | 358 | 325 | 33 | ≥20-60y | M&F combined | TV/video/DVD (average weekly time at home) | NCEP* |
| Sisson et al., 2009 [9] | USA | 2556 | 1271 | 1285 | ≥20y | M & F separately | TV/videos and computer (average hours per day in last month) | AHA/NHLBI |
| Trinh et al., 2010 [10] | Vietnam | 1244 | 1063 | 181 | 25-64y | F only | Sitting (average per day in a typical week) | Amalgamation of WHO/IDF/ATPIII^±^ |

^β^ M=male, F=female

✝ ATPIII= Adult treatment panel III, NCEP = National Cholesterol Education Programme Expert Panel, WHO = World Health Organisation, AHA/NHLBI = American Heart Association/National Heart, Lung, and Blood Institute, IDF = International Diabetes Federation

*Employed the Asia criteria for waist circumference

± required only 2 criteria to be present

**References**

1. Bankoski A, Harris TB, McClain JJ, Brychta RJ, Caserotti P, et al. (2011) Sedentary activity associated with metabolic syndrome independent of physical activity. Diabetes Care 34: 497-503.

2. Bertrais S, Beyeme-Ondoua JP, Czernichow S, Galan P, Hercberg S, et al. (2005) Sedentary behaviours, physical activity, and metabolic syndrome in middle-aged French subjects. Obes Res 13: 936-944.

3. Chang PC, Li TC, Wu MT, Lui CS, Li CI, et al. (2008) Association between television viewing and the risk of metabolic syndrome in a community-based population. BMC Public Health 8: 193.

4. Chen X, Pang Z, Li K (2009) Dietary fat, sedentary behaviours and the prevalence of the metabolic syndrome among Qingdao adults. Nutr Metab Cardiovasc Dis 19: 27-34.

5. Dunstan DW, Salmon J, Owen N, Armstrong T, Zimmet PZ, et al. (2005) Associations of TV viewing and physical activity with the metabolic syndrome in Australian adults. Diabetologia 48: 2254-2261.

6. Ford ES, Kohl HW, Mokdad AH, Ajani UA (2005) Sedentary behaviour, physical activity, and the metabolic syndrome among U.S. adults. Obes Res 13: 608-614.

7. Gao X, Nelson ME, Tucker KL (2007) Television viewing is associated with prevalence of metabolic syndrome in Hispanic elders. Diabetes Care 30: 694-700.

8. Li CL, Lin JD, Lee SJ, Tseng RF (2007) Associations between the metabolic syndrome and its components, watching television and physical activity. Public Health 121: 83-91.

9. Sisson SB, Camhi SM, Church TS, Martin CK, Tudor-Locke C, et al. (2009) Leisure time sedentary behavior, occupational/domestic physical activity, and metabolic syndrome in U.S. men and women. Metab Syndr Relat Disord 7(9): 529-536.

10. Trinh OTH, Nguyen ND, Phongsavon P, Dibley MJ, Bauman AE (2010) Metabolic risk profiles and associated risk factors among Vietnamese adults in Ho Chi Minh city. Metab Syndr Relat Disord 8(1): 55-64.
