# Supplementary material for: Association of Sedentary Behaviour with Metabolic Syndrome: A Meta-Analysis
Source: PLoS One. 2012 Apr 13;7(4):e34916. doi: 10.1371/journal.pone.0034916 (PMC3325927; doi:10.1371/journal.pone.0034916)
Supplement: Table S3 — Quality assessment of the studies included in the meta-analysis. (DOC) [file pone.0034916.s004.doc]

| Source | Prospective design | Measurement of Sedentary Behaviour | | | Objective measure of metabolic syndrome | Adjusted for 2 or more demographic, biological or behavioural confounders ✝ | Adjusted for physical activity | Adjusted for body composition |
| --- | --- | --- | --- | --- | --- | --- | --- | --- |
|  |  | Reliability of SR | Validity of SR | Objective measure |  |  |  |  |
| Bankoski et al., 2011 | No | N/A | N/A | Yes | Yes | Yes | Yes | Yes |
| Bertrais et al., 2005 | No | No | No | N/A | Yes | Yes | Yes | No |
| Chang et al., 2008 | No | No | No | N/A | Yes | Yes | Yes | No |
| Chen et al., 2008 | No | No | No | N/A | Yes | Yes | No | No |
| Dunstan et al., 2005 | No | No | Yes | N/A | Yes | Yes | Yes | No |
| Ford et al., 2005 | No | No | No | N/A | Yes | Yes | Yes | No |
| Gao et al., 2007 | No | No | No | N/A | Yes | Yes | Yes | Yes |
| Li et al., 2006 | No | No | No | N/A | Yes | Yes | Yes | Yes |
| Sisson et al., 2009 | No | No | No | N/A | Yes | Yes | Yes | Yes |
| Trinh et al., 2010 | No | No | No | N/A | Yes | Yes | No | No |

**Table S3. Quality assessment of the studies included in the meta-analysis.**

Note: Every YES was worth one point, except for objective measure which was worth two points. This was because 2 points were available against self-report instruments (1 for reliability and 1 for validity).

✝other than PA or body composition
