# Supplementary material for: Association of Sedentary Behaviour with Metabolic Syndrome: A Meta-Analysis
Source: PLoS One. 2012 Apr 13;7(4):e34916. doi: 10.1371/journal.pone.0034916 (PMC3325927; doi:10.1371/journal.pone.0034916)
Supplement: Table S4 — Results of the overall meta-analysis, sub-group and sensitivity analysis. (DOC) [file pone.0034916.s005.doc]

**Table S4. Results of the overall meta-**analysis, sub-group and sensitivity analysis.

|  | **Subgroups** | **No studies** | **No participants** | **No with MetS** | **OR** | **95% CI** | **P value** | **I^2^ (%)** | **Interaction p value** |
| --- | --- | --- | --- | --- | --- | --- | --- | --- | --- |
| **Overall** |  | 10 | 21393 | 5585 | 1.73 | 1.55 to 1.94 | **<0.0001** | 0 | - |
| **Sub-groups** |  |  |  |  |  |  |  |  |  |
| Sex | Males | 5 | 8557 | 2217 | 1.54 | 1.28 to 1.85 | **<0.0001** | 0 | 0.24 |
|  | Females | 6 | 10037 | 2053 | 2.07 | 1.70 to 2.52 | **<0.0001** | 19.3 |  |
| Quality | Lower (<=3) | 5 | 10336 | 2132 | 1.76 | 1.52 to 2.04 | **<0.0001** | 0 | 0.59 |
|  | Higher (>3) | 5 | 11057 | 3453 | 1.67 | 1.37 to 2.08 | **<0.0001** | 20.6 |  |
| Metabolic Syndrome definition | ATPIII | 3 | 4537 | 2267 | 1.56 | 1.13 to 2.17 | **0.01** | 41.5 | 0.37 |
|  | NCEP | 5 | 9631 | 1984 | 1.70 | 1.51 to 2.06 | **<0.0001** | 0 |  |
|  | WHO | 1 | 6162 | 1153 | 1.84 | 1.41 to 2.40 | **<0.0001** | - |  |
|  | Combination | 1 | 1063 | 181 | 1.87 | 1.17 to 2.99 | **0.01** | - |  |
| Measure of sedentary behaviour | TV viewing | 4 | 9487 | 2286 | 1.79 | 1.52 to 2.11 | **<0.0001** | 0 | 0.22 |
|  | TV and computer | 3 | 8016 | 2153 | 1.85 | 1.52 to 2.24 | **<0.0001** | 0 |  |
|  | Sitting time | 1 | 1063 | 181 | 1.87 | 1.17 to 2.99 | **0.01** | - |  |
|  | Accelerometer | 1 | 1367 | 665 | 1.16 | 0.77 to 1.74 | **0.48** | - |  |
|  | TV, computer and reading | 1 | 1460 | 300 | 1.52 | 1.01 to 2.29 | **0.05** | - |  |
| Country income | USA, France, Australia and Taiwan | 8 | 18689 | 5104 | 1.74 | 1.54 to 1.97 | **<0.0001** | 0 | 0.96 |
|  | China and Vietnam | 2 | 2704 | 481 | 1.66 | 1.22 to 2.26 | **<0.001** | 0 |  |
| **Sensitivity analysis** |  |  |  |  |  |  |  |  |  |
| Studies that adjusted for physical activity |  | 8 | 18689 | 5104 | 1.73 | 1.54-1.97 | <0.0001 |  |  |
