# Supplementary material for: Association of Sedentary Behaviour with Metabolic Syndrome: A Meta-Analysis
Source: PLoS One. 2012 Apr 13;7(4):e34916. doi: 10.1371/journal.pone.0034916 (PMC3325927; doi:10.1371/journal.pone.0034916)
Supplement: Table S5 — Summary of physical activity assessment in the studies that controlled for physical activity. (DOC) [file pone.0034916.s006.doc]

**Table S5. Summary of physical activity assessment in studies that controlled for physical activity.**

| **Source** | **Sedentary behaviour measure** | **Physical activity measure** | **How physical activity is controlled for** |
| --- | --- | --- | --- |
| Bankoski et al., 2011 (22) | Accelerometer (time spent at <100cpm) | Accelerometer (Active minutes were defined as ≥100 cpm) | Average count value during active minutes was added to final model (model 4) by itself. Additional analysis adjusted for minutes of moderate-vigorous physical activity in model 4 instead of the above. |
| Bertrais et al., 2005 (23) | TV and computer (average daily time at home) | Modifiable Activity Questionnaire assessed leisure time physical activity (LTPA). LTPA subjects were asked to report all activities performed at least 10 times for 10 minutes each session during leisure time over the past 12 months. Detailed information was collected about the frequency and duration of each activity reported. Using published compendiums METs were assigned to each leisure activity. Hours per week for all activities of an intensity category (<3, 3 to 6,>6 METs) were summed for each participant. Subjects were classified into three groups of LTPA: 1) insufficient activity, no LTPA reported or some LTPA but below level 2; 2) moderate activity, >=150 min/wk of LTPA >=3 METs but below level 3; and 3) vigorous activity, >=60 min/wk of LTPA >6 METs during >=20 minutes per session. | Leisure time PA level (insufficient, moderate, vigorous) added in a single multivariate model alongside other potential confounders. |
| Chang et al., 2008 (24) | TV (average daily hours) | Leisure and occupational activity were assessed. LTPA of a participant was determined by the different kinds of physical activities each person participated in, the average time spent on this activity per occasion and the total time per week the person spent performing each activity during the past year. METs were assigned to each kind of activity and then each participant's leisure-time activity was calculated by the formula of activity: MET× averaged duration of spending on this activity (hr) × total number of times a week. The occupational activity status was determined by 4 levels according to the degree of manual labour involved: seated most of the time, light, moderate, and heavy activity and then assigned a MET to each. The occupational activity status was then calculated using the formula: manual labour intensity level × working time a day (hr) × working days a week. LTPA dichotomised as <19.5 or ≥19.5 MET-hours per week. Occupational activity was categorised by tertiles of number of MET hours per week: <40, 40-83, >83. Total activity: sum of LTPA and occupational activities then divided into three categories: <54, 54-103.3, >103.3 MET-hr per week. | Occupational activity status was included as a covariate in the final model alongside age, level of education, and household income as these were significant in the univariate analyses. |
| Dunstan et al., 2005 (26) | TV/videos (total time in previous week) | The validated Active Australia Survey Questionnaire was used to ascertain participants’ frequency and duration of physical activity during the previous week. This includes items about walking for recreation or transport, ‘other’ moderate activity and vigorous activity. Total physical activity time was calculated as the sum of the time spent walking (if continuous and ≥10 min), the time spent doing other moderate-intensity activities, plus double the time spent participating in vigorous physical activity (to account for the higher volume of energy expenditure per unit time that is associated with vigorous activities). Total physical activity categories were created to reflect current recommendations: those meeting the current public health PA guidelines (≥2.5 h per week) and those not meeting the public health guidelines (0–2.49 h per week). Participants were classified as meeting the public health guidelines through walking plus moderate-intensity physical activity only (≥2.5 h per week), through vigorous activity only (≥1.0 h per week) or as not meeting these guidelines. | Total physical activity (h/week) was included in a single multivariate model alongside age, education, family history of diabetes, cigarette smoking, and dietary covariates. |
| Ford et al., 2005 (27) | TV/videos and computer (typical day in last month outside of work) | Participants were asked to report the frequency and the duration that they spent participating in 43 leisure-time physical activities of moderate or vigorous intensity. Physical activities of light intensity were not reported. Participants could also report information for up to three additional activities of moderate or vigorous intensity. Time spent doing moderate tasks around the home or yard was also included. For each activity, the weekly number of minutes spent in that activity was calculated. Total minutes per week spent performing the activities were calculated by summing the weekly minutes for each activity. We divided the total weekly minutes into three categories: 0, <150, and ≥150 min/wk of moderate or vigorous physical activity. | Physical activity (three categories) was entered into the final model by itself. |
| Gao et al., 2007 (28) | TV (average hours per day in past week) | Physical activity was estimated with a modified version of the Harvard Alumni Physical Activity Questionnaire. Participants were asked for the number of hours per day (mean of a regular weekday and a regular weekend day) spent sleeping, sitting, and engaged in light, moderate, and heavy (vigorous) activities. The time spent in each of these  activity categories was multiplied by the weighting factors 1.0, 1.1, 1.5, 2.4, and 5.0, respectively, and then summed to create a physical activity score. The cutoff score of 29 was considered conservative for sedentary activity, assuming 8 h of sleep, 8 h of sitting, and 8 h of light activity | Physical activity (by quartiles) was entered into a single multivariate model along with age, sex, ethnicity, BMI, education, household arrangement, current alcohol use, total energy intake, dietary variables and activities of daily living. |
| Li et al., 2006 (29) | TV/video/DVD (average weekly time at home) | Physical activity was assessed using a 3-day activity record. Two of the days could be any day of the week, but the third day had to be either a Saturday or a Sunday. The record divided the day into 96 periods of 15 min. The subjects were instructed to quantify their activity, on a scale of 1–9, in a diary using a list of activities and their categorical values (1–9). The approximate energy cost of each activity was derived from a variety of sources. The approximate energy cost for each category (in kcal/kg/15 min) was used to calculate the daily energy expenditure (DEE). The daily score was calculated by multiplying the amount of time spent on specific daily physical activities by the energy cost per physical activity. The DEE was calculated from the mean of the three summed daily scores. The DEE score should be viewed as a representation, not a direct measure, of energy expenditure. | Categories of DEE were defined by quartiles. The least active group consisted of subjects below the 25^th^ percentile for DEE (44.30 kcal/kg/day or less). Subjects within the 25th to 74th percentile of DEE (44.31–54.10 kcal/kg/day) were categorized as moderately active, and those in the upper quartile (greater than 75th percentile; 54.11 kcal/kg/day or greater) were categorized as the most active. In order to compare the roles of watching TV and DEE in the metabolic syndrome, they were included both separately and simultaneously in the same logistic regression model. All models were adjusted for gender, age and body mass index. |
| Sisson et al., 2009 (30) | TV/videos and computer (average hours per day in last month) | Usual occupational/domestic activity (UODA) was assessed by asking ‘Which of the 4 sentences best describes your usual daily activities?’ Response categories included “sit during the day and do not walk about very much,” “stand or walk about quite a lot during the day but do not have to lift or carry things very often,” “lift or carry light loads or have to climb stairs or hills often,” and “heavy work or carries heavy loads.” Self-reported participation in moderate and vigorous physical activity were categorized as meeting or not meeting current physical activity recommendations defined as at least 150 minutes per week of leisure-time moderate-to-vigorous physical activity. | UODA was included in the first model along with age. Model 2 included UODA, age and other covariates.  Sufficient physical activity (yes vs. no ≥150 min  per week of moderate-to-vigorous physical activity) was added by itself in the third and final model. |
